# Early Identification, Accurate Diagnosis, and Treatment of Silicosis

**DOI:** 10.1155/2022/3769134

**Published:** 2022-04-25

**Authors:** Tian Li, Xinyu Yang, Hong Xu, Heliang Liu

**Affiliations:** School of Public Health, Hebei Key Laboratory for Organ Fibrosis Research, North China University of Science and Technology, Tangshan 063210, Hebei, China

## Abstract

Silicosis is a global problem, and it has brought about great burdens to society and patients' families. The etiology of silicosis is clear, preventable, and controllable, but the onset is hidden and the duration is long. Thus, it is difficult to diagnose it early and treat it effectively, leaving workers unaware of the consequences of dust exposure. As such, a lack of details in the work history and a slow progression of lung disease contribute to the deterioration of patients until silicosis has advanced to fibrosis. These issues are the key factors impeding the diagnosis and the treatment of silicosis. This article reviews the literature on the early identification, diagnosis, and treatment of silicosis as well as analyzes the difficulties in the diagnosis and the treatment of silicosis and discusses its direction of future development.

## 1. Introduction

Silicosis is caused by the inhalation of respirable crystalline silica (RCS) dust which, over time, leads to progressive, irreversible, and fatal inflammation and fibrosis in the lungs [1]. Although the cause of silicosis is undisputed, millions of workers worldwide continue to be exposed to hazardous levels of RCS [2]. The condition is preventable; however, no specific treatment exists, although a small proportion of patients may receive a lung transplant [1]. Furthermore, the failure to recognize and control the risk associated with silica exposure in contemporary work practices, such as sandblasting denim jeans and manufacturing artificial stone benchtops, has led to the reemergence of silicosis worldwide [3]. The health hazards related to crystalline silica exposure are not only limited to silicosis; they include other idiopathic diseases such as tuberculosis, autoimmune diseases, lung cancer, pulmonary alveolar proteinosis, sarcoidosis, and idiopathic pulmonary fibrosis [3, 4]. This review outlines the most recent advances in understanding the mechanisms, diagnosis, and treatment of silicosis.

## 2. Clear Causes and Unresolved Mechanisms of Silicosis

### 2.1. Cytotoxic Effects of Silica Particles

It is well documented that the inhalation of silica particles is an environmental and occupational cause of silicosis, a type of pneumoconiosis [5]. The diverse physiochemical properties, including size, morphology, polymorphism, porosity, and surface, determine the toxicity of the silica particles. Silica particles are defined as “respirable” when they are less than 5 *μ*m in diameter, which is small enough to reach the distal airways and alveoli [5]. Furthermore, crystalline silica particles are the leading cause of occupational respiratory disease and are generally considered more toxic than amorphous silica particles [3]. The most toxic and common form of crystalline silica is *α*-quartz [6]. Recently, nearly free silanols (NFS) on the surface of quartz particles were reported as the critical molecular moieties responsible for the toxicity of silica particles [7]. High NFS concentrations are associated with increasing hemolytic potential and IL-1*β* generation, but the different toxicities of the different classes of amorphous silica particles remain poorly understood [8].

### 2.2. Recognition of Silica Particles by Macrophage Scavenger Receptors

The recognition and internalization of inhaled silica particles by alveolar macrophages in the lungs is the first critical step in initiating lung inflammation and normal immune function, both of which are regulated by macrophage scavenger receptors (SRs) such as SR-AII and macrophage receptor with collagenous structure (MARCO) [9]. A recent study has reported that SR-B1 specifically recognizes amorphous and crystalline silica particles, but not titanium dioxide nanoparticles, latex nanoparticles, or monosodium urate crystals, whereas SR-B1-mediated recognition of silica is associated with caspase-1-mediated inflammatory responses in mouse macrophages and human peripheral blood monocytes [10]. NOD-like receptor family, pyrin domain containing protein 3 (NLRP3) can activate caspase-1, which cleaves precursor cytokines that secrete interleukin (IL)-1*β*, IL-18, and IL-33 [11]. In macrophages and epithelial cells, silica-activated NLRP3 inflammasomes consisting of NLRP3, apoptosis-associated speck-like protein containing a CARD (ASC), and caspase-1 are essential for the development of silicosis [12, 13]. Silica also induced stimulator of interferon genes (STING)-dependent reactive oxygen species (ROS) production, cell death, and self-DNA release, leading to the type I interferon (IFN) response [14].

Toll-like receptors (TLRs), as pattern recognition receptors of damage-associated molecular pattern (DAMP), have been identified as critical mediators of pulmonary inflammation and fibrosis [15]. Silica particle inhalation activates TLR4 and receptor activator of nuclear factor kappa-B ligand (RANKL) signaling pathways in lung macrophages, thus inducing lung inflammation and the proteolytic phenotype of macrophages and osteoclasts in the lungs and bones [16]. Silica-induced lung inflammation, as the major cause of systemic inflammation, may disrupt the functions of extrapulmonary organs in rats exposed to long-term silica inhalation [17], which may partly contribute to our understanding of the relationship between silicosis and autoimmunity [18]. The extrathoracic manifestation of silicosis in the liver, spleen, and bone marrow [19] can be explained by the lymphangitic spread of pulmonary macrophages; however, there is still no definite and reasonable explanation.

### 2.3. Multiple Omics in Silicosis

Multiple omics approaches, including genomics, epigenomics, transcriptomics, proteomics, and metabolomics, have bridged underlying molecular alterations with silicosis progression [20]. Lung tissues, sera, peripheral blood leukocytes, and bronchoalveolar lavage fluids (BALFs) collected from silicotic patients or animals and *in vitro* models (such as silica- or TGF-*β*1-induced macrophages or fibroblasts) have been applied in omics approaches to screen diagnostic biomarkers, explore new mechanisms, and reveal potential therapeutic targets [21–25]. Single-cell RNA sequencing (scRNA-seq) provides a robust and unbiased survey of the transcriptome that is comparable to bulk RNA sequencing, while preserving cellular heterogeneity information has contributed to impressive advancements, including the discoveries of the pulmonary ionocyte and the profibrotic macrophage population in pulmonary fibrosis [26]. Emerging data from scRNA-seq analysis have provided novel insights on the dysfunction of alveolar type II progenitor epithelial cells and the diversity of mesenchymal cells within the fibrotic lung [27]. Spatial transcriptomics can extend and complement scRNA-seq studies and allow better characterization of the physiological interactions between cell types as well as their alterations in respiratory diseases, thereby providing key insights to understand their physiopathology [27, 28].

Unfortunately, most of these studies involve bolus exposure of mice to silica, followed by the evaluation of various pulmonary parameters and the completion of various tests, including lung function, bronchoalveolar lavage fluid (BALF) tests, serum tests, collagen deposition, and myofibroblast differentiation at selected times after exposure. However, the formation of fibrotic nodules indicates the irreversibility and progression of pulmonary fibrosis. Thus, there are still unsolved issues in relation to the development and the progression of silicosis. First, how can one accurately judge the line of overload of lung dust burden, which is needed to understand how much silica particle inhalation, can induce the development of silicosis? Second, why does pulmonary fibrosis continue to progress after cessation of the dust exposure, how can the lung regenerate, and can pulmonary fibrosis be reversed? These problems should be resolved to facilitate the early identification of the disease, the monitoring of disease progression, and the targeted therapies of silicosis.

### 2.4. Early Identification and Accurate Diagnosis of Silicosis

Chest X-rays (CXRs), high-resolution computed tomography (HRCT), pulmonary function tests (PFT), and health and exposure questionnaires are the major methods for the respiratory surveillance of workers exposed to RCS. However, these methods cannot detect the disease until it has significantly progressed [29]. Although it has been reported that CXR cannot reliably detect occupational lung diseases [30], it is still recommended in the diagnosis of silicosis by the International Labour Organisation (ILO) Classification System [31]. Poor-quality chest radiographs, physicians' inability to recognize the disease, and the differential latency of various types of occupational lung diseases make it difficult to detect and estimate the burden of silicosis [32, 33]. HRCT is more specific and sensitive than CXR in the early evaluation and prediction of the progression of pneumoconiosis, especially in detecting early parenchymal changes, emphysema, and pleural thickening, but it is not recommended by the International Classification of HRCT for Occupational and Environmental Respiratory Diseases (ICOERD) because of the associated high cost, radiation exposure, and low accessibility [31]. On the other hand, further studies of HRCT are warranted because it would enable a more comprehensive correlation between the pathologic findings and the clinically-relevant imaging results [34].

For the rapid and early detection of pneumoconiosis, deep convolutional diagnosis approaches have been applied to a pneumoconiosis radiograph dataset to obtain high accuracy in pneumoconiosis detection [35]. Support vector machine (SVM)-based computer-aided silicosis diagnosis could recognize silicosis-associated opacity in several candidate regions for the radiologist's reference [36]. Thus, artificial intelligence (AI)-enabled radiology tools stand to fill the need for regulatory compliance in pneumoconiosis screening, while offering a labor-saving solution to physician workflow issues and enhancing patient safety [37].

Lung biopsy should be avoided unless absolutely necessary for another reason because surgical manipulation associates with unfavorable repercussions [38]. However, lung biopsy is the only way to arrive at an accurate diagnosis when there is no occupational exposure history, disagreement between CXR and HRCT imaging results, and atypical presentations cause physicians to consider other differential diagnoses [39]. There is ongoing debate about precisely when to perform these tests and which type of biopsy should be performed in different situations [40, 41].

The guidelines for idiopathic pulmonary fibrosis recommend that surgical lung biopsy (SLB) be carried out when there is no relevant clinical manifestation and low confidence in the establishment of a diagnosis [42], with the justification that it provides the most informative tissue samples when clinicians are faced with diagnostic uncertainty [40]. Transbronchial lung cryobiopsy (TBLC), compared to SLB, appears to be safer, more accurate, and more useful because it provides meaningful information in the context of the multidisciplinary discussion of cases [41]. Transbronchial lung biopsy (TBLB) is not recommended because the specimen size is small and the specimen is susceptible to damage, thereby affecting the pathological diagnosis [43]. Patients undergoing percutaneous puncture biopsy have a high probability of pneumothorax, but CT-guided percutaneous puncture lung biopsy also has positive significance for the auxiliary diagnosis and the differential diagnosis of pneumoconiosis [44].

## 3. Treatment for Silicosis

Due to the unavailability of an early diagnostic protocol, the treatment of silicosis is mostly limited to basic research, and therapeutic interventions for silicosis are still limited. Large volume whole-lung lavage is often used at an early category of silicosis to improve chest tightness, chest pain, shortness of breath, and other related symptoms. A recent study reported significant radiological improvement in patients with artificial stone-associated silicosis that may have better long-term outcomes in terms of symptomatology, functional capacity, lung function, and mortality [45]. Furthermore, successful exercise progression maximizes improvements and sustained treatment effects favor those with milder interstitial lung diseases [46].

Pirfenidone and nintedanib, approved by the U.S. Food and Drug Administration (FDA) for idiopathic pulmonary fibrosis, have been well documented in silicotic models [47, 48]. Tetrandrine, which has been approved to treat silicosis in China [49], can inhibit lung inflammation and lung fibrosis to improve pulmonary function [50]. In recent years, herbal compounds have been documented to trigger the generation of ROS, suppress the activation of inflammasomes, and exhibit anti-inflammatory and antifibrotic effects [51]. Clinical trials have shown that patients' lung function, quality of life, and exercise ability have significantly improved after treatment with traditional Chinese medicines [52].

Lung transplantation is the main modality for the treatment of end-category silicosis, and it can help patients with end-category fibrosis to live longer. However, lung transplantation is expensive, difficult, and risky, and the median survival after transplantation is relatively short (approximately 6–7 years) [3]. In addition, long-term oral antirejection immunosuppressants are required. Studies have shown that the 3-year survival rate of silicosis patients after lung transplantation is approximately 76% [53].

Other potential drugs have been reported to inhibit silica-induced pulmonary inflammation and fibrosis in silicotic models such as anticytokines (anakinra, anti-IL-17 antibody, and anti-IL-9 antibody), antioxidants (N-acetylcysteine, corticosteroids, and dexamethasone), agents influencing the autophagic-lysosome system (imipramine, dioscin, and rapamycin), agents increasing cAMP levels, and microRNAs [5]. Mesenchymal stromal cells (MSCs) and MSC-derived extracellular vesicles (EVs) possess immunoregulatory and reparative abilities, which modulate innate and adaptive immunity and have shown great therapeutic potential in silicosis [54]. Presently, there are no large, randomized, and placebo-controlled clinical trials to assess the safety and the efficacy of these drugs in silicosis, and thus, their safety and efficacy remain to be determined.

## 4. Summary

When silicotic nodules and collagen deposits are visible in rodent silicotic models, treatment is likely to be ineffective. Clinicians often assess myofibroblast differentiation, inflammation, macrophage activation, and epithelial-mesenchymal transition to evaluate the antifibrotic effects of potential drugs [16, 55–57]; however, here too, treatment is likely to be ineffective. Multiomics approaches, in conjunction with an established silicotic model, can be used to study the mechanism of silicosis and to identify potential biomarkers of the disease [21, 22, 58–61]. Unfortunately, our understanding of omics is relatively poor, and diagnostic markers should comprise a large group of biomarkers reflecting the different pathological states of the disease.

In conclusion, silicosis still poses a threat to the health of many individuals worldwide. However, there is a lack of information on effective early prevention, early diagnosis, and timely drug treatment. Thus, it is important to explore additional pathological mechanisms that might be associated with silicosis and to identify novel early diagnostic and therapeutic modalities to improve the prognosis of silicosis patients worldwide.
